# Partial Compensation of IL‐17 Production by Vγ1 T Cells in the Absence of Vγ4 and Vγ6 T Cells

**DOI:** 10.1002/eji.70061

**Published:** 2025-09-20

**Authors:** Ziqing Wang, Tao Yang, Federico Lupo, Lara‐Marie Behrens, Anika Janssen, Seth B. Coffelt, Immo Prinz, Inga Sandrock, Sarina Ravens

**Affiliations:** ^1^ Institute of Immunology Hannover Medical School Hannover Germany; ^2^ School of Cancer Sciences University of Glasgow Glasgow UK; ^3^ Cancer Research UK Scotland Institute Glasgow UK; ^4^ Institute of Systems Immunology Hamburg Center for Translational Immunology University Medical Center Hamburg‐Eppendorf Hamburg Germany; ^5^ Cluster of Excellence RESIST (EXC 2155) Hannover Medical School Hannover Germany

**Keywords:** γδ T cells, Development, IL‐17, T cell receptor

## Abstract

γδ T cells are unconventional T cells that group into different subsets based on the usage of variable γδ T cell receptor (TCR) gene segments, body location, and functionality. γδ T cells that secrete the proinflammatory cytokine interleukin 17 (IL‐17) predominantly express a Vγ4^+^ or Vγ6^+^ γδ TCR. The biology and the importance of the γδ TCR of IL‐17‐producing γδ T cells are not well understood. Here, we investigated the IL‐17 production capability of γδ T cells in mice deficient for Vγ4^+^ and Vγ6^+^ γδ TCRs using flow cytometry, TCR‐seq, and single‐cell transcriptomics. Our data show that Vγ1 T cells only partially compensate for the loss of IL‐17^+^ Vγ4 and Vγ6 T cell subsets in lymphoid and nonlymphoid tissues. They develop pre‐ and postnatally and were predominantly detectable in their physiological body habitats. Collectively, the data underscore the nonredundant roles of Vγ4⁺ and Vγ6⁺ subsets in IL‐17‐mediated immunity.

## Graphical abstract

In the absence of Vγ4⁺ and Vγ6⁺ γδ TCRs, Vγ1 T cells only partially compensate for IL‐17 production. These Vγ1 T cells develop pre‐ and postnatally and show enhanced γδ17 gene signature, but are absent in skin, highlighting the nonredundant role of Vγ4⁺ and Vγ6⁺ subsets in IL‐17‐mediated immunity.

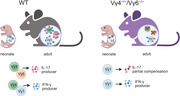

## 1 Introduction

γδ T cells play a crucial role in the immune system as rapid responders at the interface of innate and adaptive immunity, capable of recognizing stress signals and pathogens without the need for classical antigen presentation. γδ T cells act as a defense against pathogens, as proinflammatory cells, or as mediators of tissue homeostasis. In general, murine γδ T cells are divided into two main functional subsets: those that produce the cytokine IL‐17 (γδ17) and those that secrete IFN‐γ (γδ1). However, certain γδ T cells also exhibit functional plasticity. For example, subsets in the gut can co‐produce IL‐17 and IFN‐γ [1], and inflammatory stimuli can induce IFN‐γ production by γδ17 cells [2, 3, 4]. Furthermore, γδ T cells can exhibit a variety of functional phenotypes, such as interacting with other immune cells and producing growth factors under certain circumstances [5].

Moreover, certain subsets develop during the prenatal period, at which point they acquire effector functions, including the capacity for rapid cytokine release, prior to leaving the thymus [6]. The genomic organization of the γδ T cell receptor (TCR) locus suggests that rearrangement processes of the variable (V) genes of the TCR might be developmentally regulated, which could explain the ordered appearance of distinct TCRs [7, 8, 9]. Overall, the time of appearance is associated with the Vγ‐segment usage of the γδ T‐cell receptor (TCR), location, and functionality [10, 11]. From the embryonic day 15 on the development starts with murine IFN‐γ^+^ Vγ5 T cells that seed to the epidermis (known as DETCs) [12]; followed by Vγ6 T cells that become IL‐17 producers and populate different tissue sites, and by a fraction of Vγ4 T cells that have also the potential to secrete IL‐17 shortly after the Vγ6 T cells [12]. In the perinatal and postnatal period, Vγ7 T cells home to the intraepithelial compartment of the gut, and Vγ1 and Vγ4 T cells become naïve CD27^+^ circulating cells in lymphoid and other organs such as the liver, where they further differentiate into IFN‐γ^+^ γδ T cells upon activation [10, 14]. In addition, CD27^+^ γδ T cells can convert into highly cytotoxic, anticancer CD27^+^ Ly6C^+^ γδ T cells [15].

The default programming of IL‐17 production that is evident prior to TCR expression in thymic Vγ6^+^ γδ T cell progenitor cells is one key factor in determining function [13, 16]. It is unclear whether this is linked to the hematopoietic origin of fetal‐derived cells, as it has been described for Vγ5 T cells [17]. Furthermore, TCR signals play a pivotal role in the functional differentiation and the maintenance of γδ T cell subsets. It has been proposed that strong TCR signals lead to the development of an IFN‐γ‐secreting phenotype, while weak TCR signals favor the development of IL‐17 producers [14, 18, 19, 20, 21, 22]. To illustrate, during development, the engagement of the Vγ5^+^ TCR with Skint‐1 diverts these cells away from key molecules associated with IL‐17‐producing cells, such as Sox13, RORγt, and Scart‐2, and initiates differentiation toward IFN‐γ‐secreting cells [20]. Moreover, studies on the expression of a haploinsufficient CD3 mutant in vivo, as well as on the modulation of the TCR signaling strength in developing γδ T cells in vitro, suggest that weaker TCR signals support the generation of IL‐17 producers independently of Vγ‐chain usage [19, 23]. However, the knowledge of γδTCR ligands and their role in the functional differentiation and peripheral maintenance for murine Vγ6 and Vγ4 T cells, which are the dominant IL‐17 producers, remains elusive [21]. It has been suggested that the TCRs of γδ17 cells are poly‐specific and can be activated by various small metabolic products [24, 25, 26].

To determine the implication of Vγ chain usage on the functional commitment and tissue distribution of γδ17 cells, we investigated the IL‐17 production in mice that were double deficient for Vγ4^+^ and Vγ6^+^ TCRs. To this end, we used a well‐established mouse model that is deficient in both Vγ4^+^ and Vγ6^+^ γδ T cells [27]. In these mice, Vγ1 T cells constitute the predominant population in the peripheral lymphoid organs, liver, and lung. This mouse model has been widely used to study cross‐talk between γδ T cells and other immune cells, as well as the balance between different γδ T cell subsets in immune responses. In particular, it has been employed to study the interaction of γδ T cells with B cells, and it has been shown that a deficiency of specific γδ T cell subsets has a greater impact on antibody secretion and B cell maturation, and it was presumed that Vγ1 T cells are counter‐regulated by Vγ4 T cells in this case [28, 29, 30, 31]. In this study, we examined the prevalence of Vγ1 T cells in various body regions and investigated their capacity for IL‐17 production, the TCR repertoire, and gene expression programs in lung Vγ1 T cells. In summary, the data indicate that Vγ1^+^ γδ T cells only partially compensate for the loss of IL‐17^+^ Vγ4 and Vγ6 T cell subsets in normal body habitats of Vγ1 T cells. This reinforces the view that the expressed TCR associates with IL‐17‐producing γδ T cells.

## 2 Results

### 2.1 Enrichment of IL‐17^+^ CD44^high^ Vγ1 T Cells in the Absence of Vγ4 and Vγ6 T Cells

**FIGURE 1:**
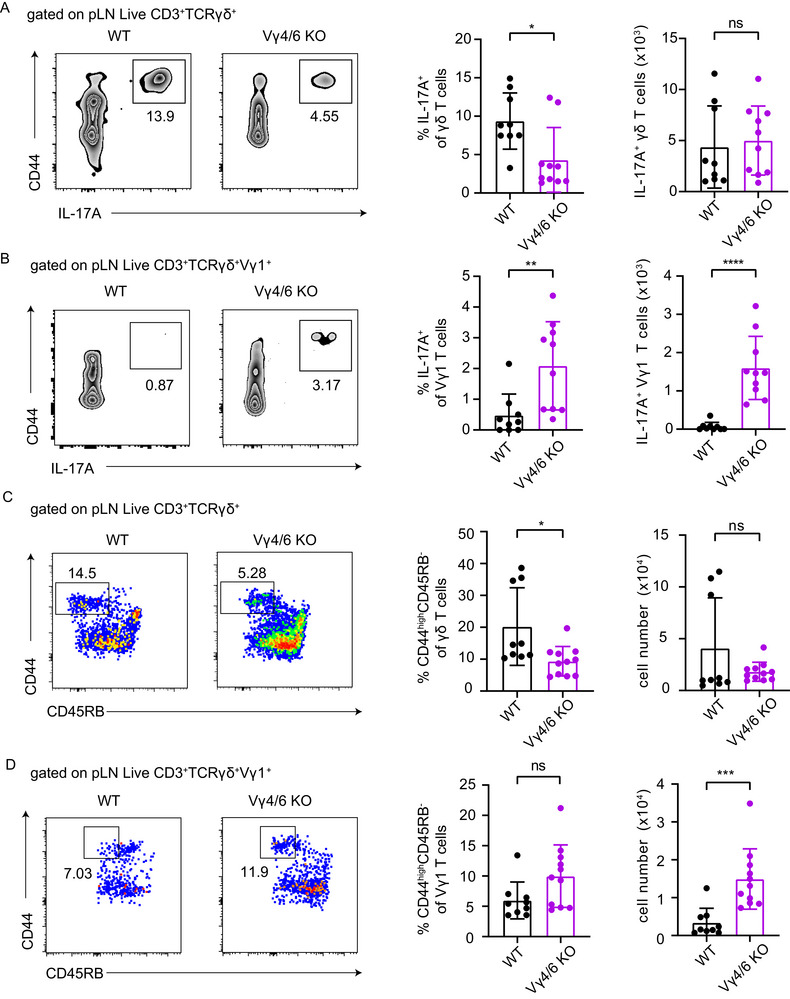
Abundance of IL‐17‐producing γδ T cells in Vγ4^−/−^/Vγ6^−/−^ adult mice. (A) Representative FACS plots and graphs demonstrate frequencies and cell numbers of IL‐17‐producing γδ T cells in the peripheral lymph nodes of WT and Vγ4^−/−^/Vγ6^−/−^ mice. (B) Representative FACS plots and graphs indicate frequencies and cell numbers of IL‐17‐producing Vγ1 T cells in WT and Vγ4^−/−^/Vγ6^−/−^ mice. Cytokine production was assessed after overnight in vitro stimulation of lymphocytes with IL‐23 and IL‐1β. (C) Representative FACS plots and graphs show frequencies and cell numbers of CD44^high^ CD45RB^−^ γδ T cells in the pLNs of WT and Vγ4^−/−^/Vγ6^−/−^ mice. (D) Representative FACS plots and graphs demonstrate frequencies and cell numbers of CD44^high^ CD45RB^−^ Vγ1 T cells in the pLNs of WT and Vγ4^−/−^/Vγ6^−/−^ mice. Data are representative of three independent experiments with *n* = 3 or 4 per group. *p*‐values were determined using an unpaired Mann–Whitney test (ns *p* > 0.05, *p < 0.05, **p < 0.01, ***p < 0.001, ****p < 0.0001). γδ T = gamma‐delta T; WT = wild‐type; KO = Vγ4^−/−^/Vγ6^−/−^.

Here, we investigated the IL‐17 production capability of γδ T cells in Vγ4^−/−^/Vγ6^−/−^ knock‐out (KO) mice, which lack Vγ4 and Vγ6 T cells. An overall increase in the frequency, but not the total cell number, of γδ T cells among CD3^+^ T lymphocytes was observed (Figure S1A,B). Moreover, the number of total pLN Vγ1 T cells was significantly higher in Vγ4^−^/^−^ / Vγ6^−^/^−^ mice (Figure S1C). Next, to study IL‐17 production by γδ T cells, peripheral lymph node (pLN) cells from wild‐type (WT) and Vγ4^−/−^/Vγ6^−/−^ mice were stimulated with the cytokines IL‐1β and IL‐23 in vitro. IL‐17A production by γδ T cells, as measured by frequency, was significantly reduced, though not completely absent, in Vγ4^−/−^/Vγ6^−/−^ mice (Figure 1A). Total cell numbers were similar among WT and Vγ4^−/−^/Vγ6^−/−^ mice (Figure 1A). Vγ1 T cells accounted for the majority (approximately 80%) of IL‐17⁺ γδ T cells in Vγ4^−/−^/Vγ6^−/−^ mice. However, some IL‐17A⁺ cells were also present in non‐Vγ1 T cell subsets (Figure S1D). Regarding Vγ1 T cells specifically, an increase in the frequency of IL‐17^+^ cells among Vγ1 T cells and in the total number of IL‐17^+^ Vγ1 T cells was evident (Figure 1B). Overall, these results suggest either homeostatic expansion or increased development of IL‐17^+^ Vγ1 T cells in the absence of Vγ4 and Vγ6 T cells.

Next, we investigated the phenotypes of γδ T cells in the Vγ4^−/−^/Vγ6^−/−^ mice and compared them with WT conditions. The expression of the surface proteins CD44 and CD45RB allows γδ T cells to be separated into two subsets: CD44^high^ CD45RB^−^, referred to as IL‐17 producers (γδ17), and CD44^int^ CD45RB^+^, referred to as type 1 immunity effector (γδ1) subsets [18]. The frequency of CD44^high^ CD45RB^−^ γδ T cells, but not the cell numbers, was lower in Vγ4^−/−^/Vγ6^−/−^ mice than in WT conditions (Figure 1C). Consistent with IL‐17 production capability, slightly increased frequencies and numbers of CD44^high^ CD45RB^−^ Vγ1 T cells were noted in the lymphoid organs of Vγ4^−/−^/Vγ6^−/−^ mice (Figure 1D).

In summary, the data indicate that the absence of Vγ4 and Vγ6 T cells is partly compensated by elevated levels of IL‐17A produced by Vγ1 T cells in peripheral lymphoid organs.

### 2.2 TCR Repertoires of IL‐17^+^ and IL‐17^−^ Vγ1 T Cells Are Highly Similar

**FIGURE 2:**
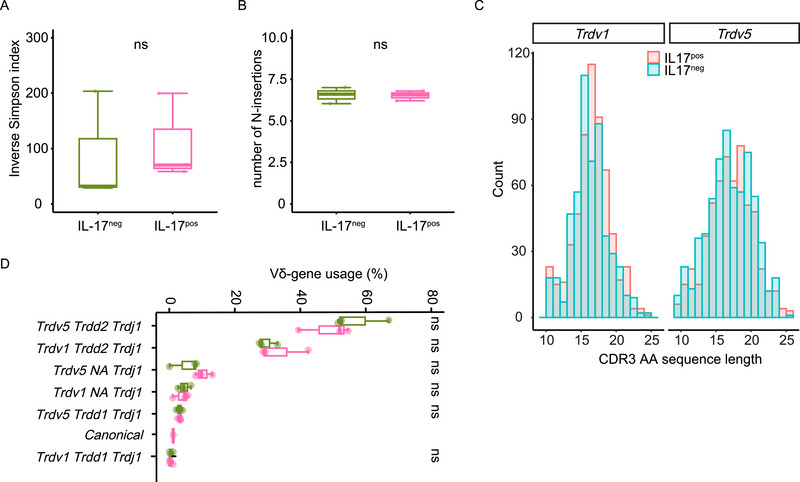
TCR repertoires of IL‐17^pos/neg^ γδ T cells do not differ in Vγ4^−/−^/Vγ6^−/−^ mice. TCR‐seq analysis of FACS‐sorted IL‐17‐secreting γδ T cells or non‐IL‐17‐secreting γδ T cells from pLNs of Vγ4^−/−^/Vγ6^−/−^ mice (three samples per group). (A) Inverse Simpson diversity indices of the *Trd* repertoire of IL‐17^neg^ γδ T cells (green) and IL‐17^pos^ γδ T cells (pink), calculated based on the variety of CDR3.nt sequences within the repertoire. (B) Number of N‐insertions in the respective subsets. (C) Distribution of CDR3 amino acid lengths in *Trdv1* and *Trdv5* clones from IL‐17^neg^ γδ T cells (blue) and IL‐17^pos^ γδ T cells (red). (D) Usage of the δ chain. Percentage of total repertoire shown for each rearrangement. Statistical analysis was performed using the Wilcoxon test (ns *p* > 0.05, *p < 0.05, **p < 0.01, ***p < 0.001, ****p < 0.0001).

Fetal‐derived γδ T cells have a restricted TCR repertoire with minimal diversity, whereas it is thought that postnatal‐derived T cells possess significantly more diverse TCR repertoires [32]. To elucidate whether IL‐17^+^ and IL‐17^−^ Vγ1 T cells exhibit similar or different TCR repertoire diversity, an mRNA‐based next‐generation sequencing analysis of δ chain sequences (TCR‐seq) was conducted on FACS‐sorted IL‐17^+^ and IL‐17^−^ γδ T cells obtained from pLNs of Vγ4^−/−^/Vγ6^−/−^ mice. The TCR repertoire diversity, as measured by the inverse Simpson index, showed no significant difference between IL‐17^+^ and IL‐17^−^ cells (Figure 2A). Moreover, similar numbers of N‐insertions (nucleotide insertions) within the CDR3 region of δ‐chain sequences were observed in both IL‐17^+^ and IL‐17^−^ cells (Figure 2B). Also, the length of the CDR3 region was found to be highly similar between IL‐17^+^ and IL‐17^−^ cells that were either *Trdv1* ^+^ or *Trdv5* ^+^ (Figure 2C). In terms of Vδ‐gene usage, approximately 50% of the sequences consist of the *Trdv5* gene segment, while 30% of the sequences consist of the *Trdv1* gene segment, regardless of cytokine production (Figure 2D). Notably, Kashani et al. [33] found a specific germline‐rearranged, canonical Vδ5Dδ2Jδ1 sequence that was restricted to IL‐17‐producing, fetal‐derived Vγ4 T cells. In this study, the Vδ5Dδ2Jδ1^+^ canonical TCR sequence was present in only one of the IL‐17^+^ samples (Figure 2D). Taken together, the TCR repertoire analysis depicts a high similarity between the TCRs of IL‐17^+^ and IL‐17^−^ Vγ1 T cells. This suggests that IL‐17 production is not associated with a specific set of Vγ1^+^ TCRs or a particular origin in early life.

### 2.3 Vγ1 T Cells Accumulate as CD44high CD45RB^−^ Cells in the Lung of Vγ4^−/−^/Vγ6^−/−^ Mice

**FIGURE 3:**
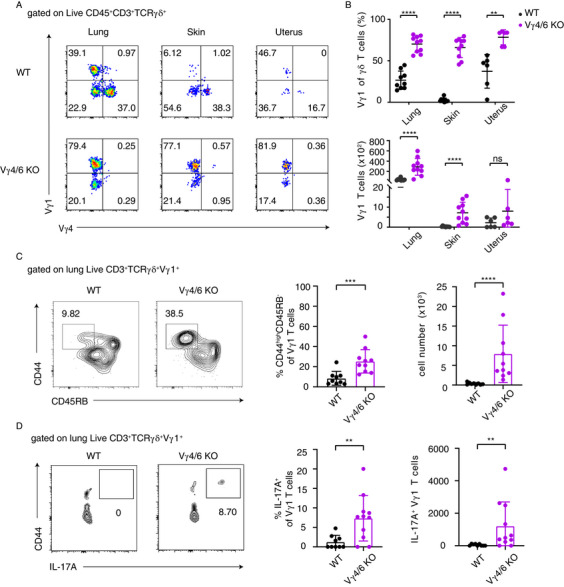
Abundance and phenotypes of Vγ1 T cells in tissues. (A) Representative FACS plots demonstrate frequencies of Vγ1 T cells in the lung (left), skin (middle), and uterus (right) of WT and Vγ4^−/−^/Vγ6^−/−^ mice. (B) Graphs show frequencies (up) and cell numbers (down) of Vγ1 T cells in the lung, skin, and uterus of WT and Vγ4^−/−^/Vγ6^−/−^ mice. (C) Representative FACS plots and graphs demonstrate frequencies and cell numbers of CD44^high^ CD45RB^−^ Vγ1 T cells in the lung of WT and Vγ4^−/−^/Vγ6^−/−^ mice. (D) Representative FACS plots and graphs demonstrate frequencies and cell numbers of IL‐17‐producing Vγ1 T cells in the lung of WT and Vγ4^−/−^/Vγ6^−/−^ mice. Cytokine secretion was assessed after overnight stimulation with IL‐23 and IL‐1β. Data are representative of two or three independent experiments with *n* = 3 or 4 per group. *p*‐values were determined using an unpaired Mann–Whitney test (ns *p* > 0.05, *p < 0.05, **p < 0.01, ***p < 0.001, ****p < 0.0001). γδ T = gamma‐delta T; WT = wild‐type; KO = Vγ4^−/−^/Vγ6^−/−^.

Since γδ T cells are a major source of IL‐17 in tissues [34], we next studied these cells in the lung, uterus, and skin of Vγ4^−/−^/Vγ6^−/−^ mice. We confirmed that the lungs and the uteruses of WT mice consist of Vγ1 T cells, Vγ4 T cells, and others (presumably Vγ6^+^), whereas Vγ1 T cells are largely absent from the skin (Figure 3A). Nevertheless, the frequency of Vγ1 T cells among the total γδ T cells was increased in all studied tissues in the absence of Vγ4 and Vγ6 T cells (Figure 3B). The absolute cell number of Vγ1 T cells was also higher in the tissues of Vγ4^−/−^/Vγ6^−/−^ mice, with the highest abundance observed in the lungs (Figure 3B).

Next, the phenotypic analyses focused on γδ T cells in the lung, given the higher number of cells present in this organ. Increased frequencies and numbers of CD44^high^ CD45RB^−^ Vγ1 T cells were evident in the lung of Vγ4^−/−^/Vγ6^−/−^ mice (Figure 3C). In addition, CD44^high^ Vγ1 T cells expressed the surface marker CD103, indicating tissue‐resident T cells (Figure S2B). Furthermore, IL‐17A production was assessed following in vitro stimulation with IL‐1β and IL‐23. An increase in IL‐17A^+^ Vγ1 T cells was observed in the lungs of Vγ4^−/−^/Vγ6^−/−^ mice (Figure 3D). However, overall IL‐17A production by total γδ T cells was significantly reduced in Vγ4‐ and Vγ6‐deficient mice (Figure S2C). To additionally explore whether the lung environment preferentially supports γδ17 differentiation, we compared the frequency and absolute number of CD44^high^ CD45RB^−^ Vγ4 T cells in the lungs and skin of only WT mice (Figure S2D). While the lung contained a greater number of total CD44^high^ CD45RB^−^ Vγ4 T cells, the frequency of these cells within the γδ T cell population was similar in the lung and skin. Notably, only CD44^high^, but not CD45RB^+^, Vγ4 T cells were evident in the skin (Figure S2D). Collectively, these data imply that, in some tissues (particularly in the lung), IL‐17‐producing Vγ1 T cells increase as tissue‐resident cells in Vγ4^−/−^/Vγ6^−/−^ mice.

### 2.4 Enhanced γδ17 Gene Expression Signatures in Lung Vγ1 T Cells from Vγ4^−/−^ /Vγ6^−/−^ Mice

**FIGURE 4:**
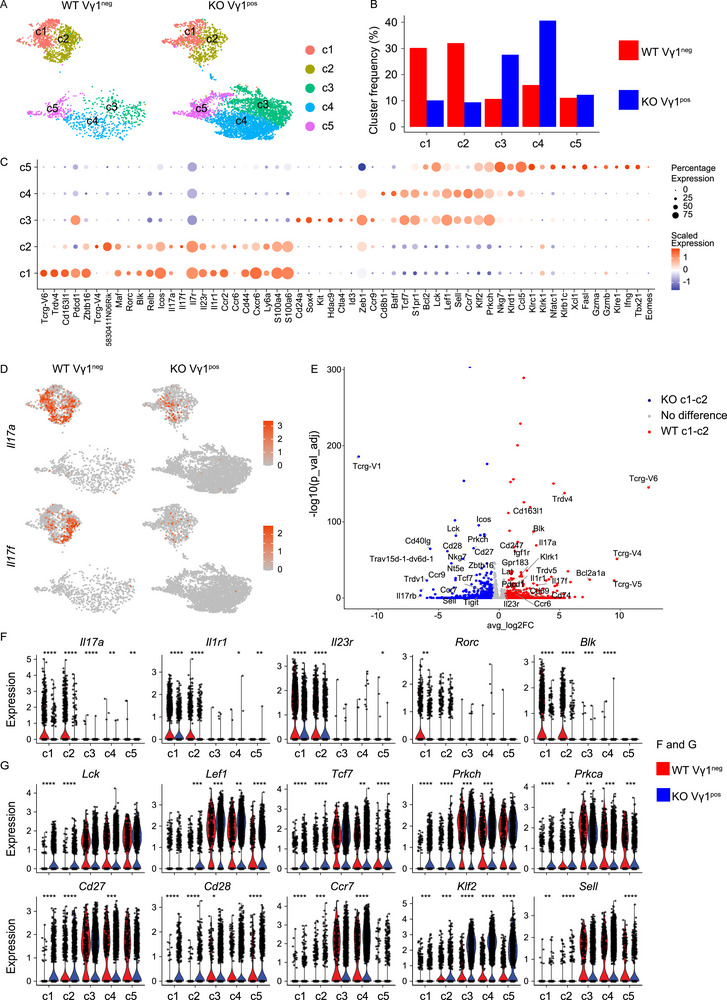
scRNA‐seq identifies γδ17 gene expression profiles in lung Vγ1 T cells. (A) UMAP representation of WT Vγ1^neg^ and KO Vγ1**^pos^** T cell transcriptomes, colored by cluster. (B) The bar plot reveals fractions (%) of absolute cell numbers from WT Vγ1^neg^ and KO Vγ1**^pos^** T cells that contribute to clusters c1‐c5. (C) Dot plots displaying average gene expression per cluster. Gene expression values were scaled to a log2 fold change (logFC). Dots are colored by average logFC and sized by the percentage of cells expressing each gene per cluster. (D) UMAPs show *Il17a* and *Il17f* expression in WT Vγ1^neg^ and KO Vγ1**^pos^** T cells. (**E**) Volcano plot shows differentially expressed genes (DEGs) among WT Vγ1^neg^ and KO Vγ1^pos^ γδ17 cluster (c1‐c2). Upregulated DEGs are identified with log2FC >0.5 and p_val_adj <0.05. (**F‐G**) Violin plots show gene expression of indicated genes in the five clusters and are divided between WT Vγ1^neg^ and KO Vγ1^pos^ T cells. Statistical significance was determined by unpaired two‐sided Wilcoxon–Mann–Whitney *U* test; (not significant, *p* > 0.05 (not shown), *p < 0.05, **p < 0.01, ***p < 0.001, ****p < 0.0001).

To investigate the molecular programs of lung Vγ1 T cells in the absence of Vγ4 and Vγ6 T cells, single‐cell RNA sequencing (scRNAseq) analysis was performed on lung Vγ1 T cells of Vγ4^−/−^/Vγ6^−/−^ mice (KO). The transcriptional profiles were then compared with those of lung non‐Vγ1 T cells (most likely Vγ4 and Vγ6 T cells) of WT mice (WT). The transcriptomes of 5253 KO Vγ1 T cells and 2191 WT non‐Vγ1 T cells were obtained and divided into five Clusters (c1‐c5) according to their differentially expressed genes (DEG) (Figure 4A; Figure S3A). Clusters c1‐c2 were overrepresented in WT lungs (Figure 4B; Figure S3B). Clusters c1 and c2 were identified as γδ17 cells and can be further subdivided into definitive‐positive cells, which express canonical type 3 genes, including *Maf*, *Rorc*, *Il17a*, and *Il17f*, and definitive‐negative cells, which lack these markers but express other type 3 genes (e.g., *Blk*, *Icos*, *Ccr2*, *S100a4*) (Figure 4C; Figure S3C,D).

Of the two γδ17 Clusters, cluster c1 was identified as Vγ6 T cells, characterized by high expression of *Tcrg‐V6*, *Trdv4* (Vδ1 chain), *Cd163l1* (encoding SCART1), and *Pdcd1* (encoding PD‐1) (Figure 4C; Figure S3D). These are established gene markers for IL‐17A^+^ Vγ6 T cells [35, 36, 37, 38, 39]. In contrast, Cluster c2 cells display high expression of *Tcrg‐V4* and *5830411N06Rik* (encoding SCART2) and have therefore been annotated as IL‐17A^+^ Vγ4 T cells [38] (Figure 4C; Figure S3D). Interestingly, KO Vγ1 T cells also cluster in c1‐c2, but do not express *Tcrg‐V4* or *Tcrg‐V6*; instead, they express *Tcrg‐V1* (Figure 4A; Figure S3D,E). Expression of γδ17 T cell‐related markers such as *Maf*, *Ccr2*, and *S100a4* suggests that the Vγ4^−/−^/Vγ6^−/−^ KO Vγ1 T cells in cluster c1‐c2 present the same γδ17 Clusters as the WT non‐Vγ1 T cells (Figure S3F).

Clusters c3‐c4 were overrepresented in the FACS‐sorted Vγ1 T cells of KO mice and were identified as IFNγ‐committed γδ (γδ1) cells because they express *Tcf7*, *Lck*, *Batf*, and *Klrd1*. These genes have been previously reported to be associated with γδ1 cells [35] (Figure 4B,C). Cluster 5 is present at similar levels in WT and Vγ4^−/−^/Vγ6^−/−^ KO mice and shows high levels of *Eomes*, *Gzma*, and *Gzmb* expression, associated with cytotoxicity (Figure 4B,C; Figure S3B,D).

Consistent with the FACS data obtained from in vitro stimulation assays, transcripts for *Il17a* and *IL17f* were detected in Vγ1 T cells from KO mice, albeit at a lower abundance, as illustrated in the feature UMAP plot (Figure 4D).

To further define transcriptional differences, DEGs analysis was performed on WT and KO cells from the γδ17 Cluster c1‐c2 (Figure 4E). As expected, *Tcrg‐V4* and *Tcrg‐V6* transcripts are significantly enriched in WT Vγ1^neg^ T cells. Importantly, KO Vγ1^+^ T cells displayed lower expression of effector genes such as *Il17a* and *Il17f*, indicating downregulated functionality. They also displayed lower expression of cytokine receptors, including *Il23r* and *Il1r1*, as compared with WT cells (Figure 4E,F). In contrast, the γδ17 Clusters of KO Vγ1 T cells showed high expression of TFs involved in TCR signaling, such as *Lck*, *Lef1*, and *Tcf7* (encoding TCF‐1) [40, 41, 42] as well as other molecules related to TCR signaling, such as *Prkch* and *Prkca* (Figure 4E,G). They also displayed high expression of the tumor necrosis factor receptor *Cd27* and immunoglobulin member *Cd28*, which act as costimulatory molecules for γδ1 T cells. They also displayed higher expression of the tissue homing markers *Ccr7*, *Klf2*, and *Sell* (encoding CD62L) (Figure 4E,G). Taken together, scRNAseq analysis revealed the RNA expression profiles of γδ17 cells in Vγ1 T cells in the absence of Vγ6 and Vγ4 T cells. These cells showed enrichment for genes associated with high TCR signaling events, setting them apart from γδ17 cells in WT conditions.

### 2.5 IL‐17^+^ Vγ1 T Cells Are Present in the Adult, Neonatal, and Fetal thymus in Vγ4^−/−^/Vγ6^−/−^ Mice

As lung Vγ1 T cells show simultaneous expression of transcriptional programs of γδ17 cells, for example, *Rorc*, and of γδ1 cells, for example, *Lck* or *Cd27*, in Vγ4^−/−^/Vγ6^−/−^ mice, we asked about the presence of c‐Maf and Rorγt‐expressing cells in the adult thymus.

**FIGURE 5:**
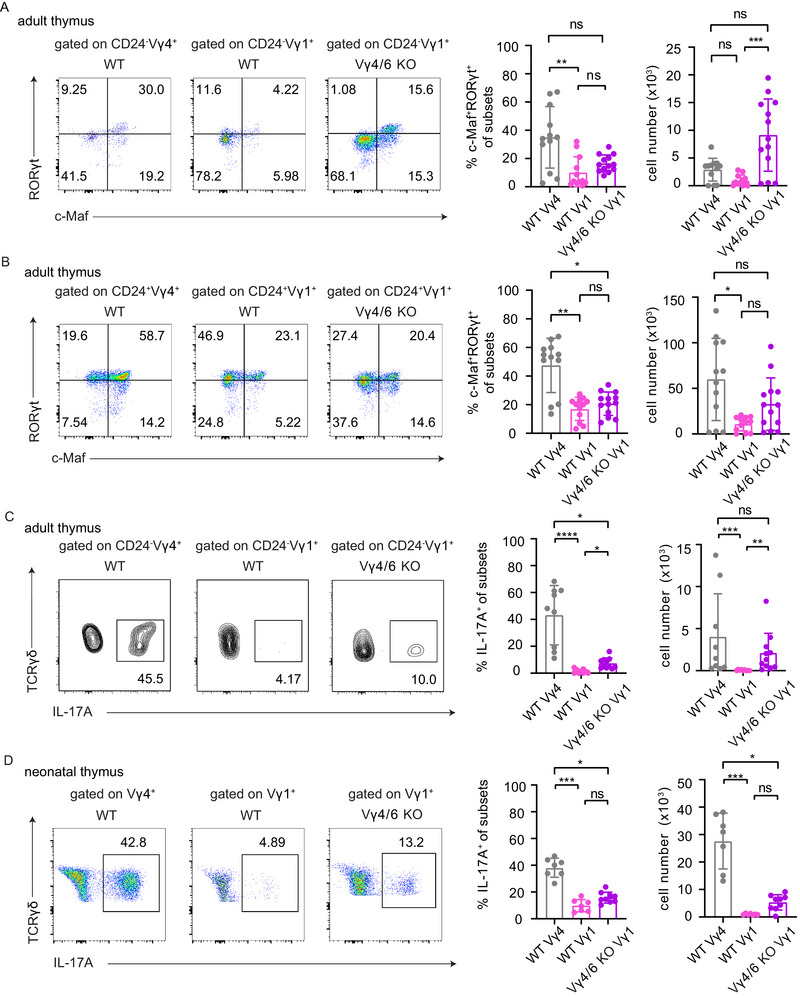
c‐Maf/RORγt expression and IL‐17 production in thymic Vγ1 T cells. (A) Representative FACS plots showing c‐Maf and RORγt expression in mature (CD24^−^) Vγ1 T cells from the thymus of adult (5–7 weeks old) WT and Vγ4^−/−^/Vγ6^−/−^ mice. WT Vγ4 T cells are shown as a reference. Quantification (right) shows the percentage and absolute numbers of c‐Maf⁺ RORγt⁺ cells among γδ T cells. (B) Representative FACS plots showing c‐Maf and RORγt expression in immature (CD24^+^) Vγ1 T cells from the thymus of adult WT and Vγ4^−/−^/Vγ6^−/−^ mice. Quantification (right) shows the percentage and absolute numbers of c‐Maf⁺RORγt⁺ cells among γδ T cells. (C) Frequencies of IL‐17A expression in mature (CD24^−^) thymic γδ T cells from adult WT and Vγ4^−/−^/Vγ6^−/−^ mice after overnight in vitro stimulation with IL‐23 and IL‐1β. Quantification (right) shows the percentage and absolute numbers of IL‐17A⁺ γδ T cells in the thymus. (D) Frequencies of IL‐17A expression in thymocytes from neonatal (day 5) WT and Vγ4^−/−^/Vγ6^−/−^ mice following in vitro stimulation with PMA and ionomycin. Quantification (right) shows the percentage and absolute numbers of IL‐17A⁺ γδ T cells in the thymus. Data are representative of two or three independent experiments, with *n* = 3–4 mice per group. Statistical analysis was performed using a nonparametric Kruskal–Wallis test with Dunn's multiple comparisons correction. (ns *p* > 0.05, *p < 0.05, **p < 0.01, ***p < 0.001, ****p < 0.0001). γδ T = gamma‐delta T; WT = wild‐type. KO = Vγ4^−/−^/Vγ6^−/−^.

In the thymus, CD24 can be employed to define immature and mature γδ T cells [35, 41]. First, thymic CD24^−^ Vγ1 T cells do express c‐Maf and RORγt in both WT and Vγ4^−^/^−^Vγ6^−^/^−^ mice. Nevertheless, the frequencies of c‐Maf^+^ RORγt^+^ cells among WT CD24^−^ Vγ4 T cells were highest (Figure 5A). For immature cells, defined as CD24^+^, the highest frequency and cell number of c‐Maf^+^ RORγt^+^ cells were observed for thymic Vγ4 T cells of WT mice (Figure 5B). There was no significant difference in the frequencies and cell numbers of c‐Maf^+^ RORγt^+^ CD24^+^ Vγ1 T cells among Vγ4^−/−^/Vγ6^−/−^ and WT mice (Figure 5B).

Next, the production of IL‐17 by CD24^−^ Vγ4 and Vγ1 T cells was assessed in the adult thymus. The data show that Vγ4 T cells in WT conditions produce IL‐17 in particular. In addition, higher levels of IL‐17 production were observed in Vγ1 T cells from Vγ4^−/−^/Vγ6^−/−^ mice than in Vγ1 T cells from WT mice (Figure 5C). A similar experiment was performed on γδ T cells in the neonatal thymus. In all conditions, IL‐17 production was evident, with Vγ4 T cells being the highest IL‐17 producers in WT neonatal thymocytes (Figure 5D; Figure S4A). In addition, the abundance of CD44^+^ cells with respect to IL‐17 secretion and the expression of the Vγ1^+^ or Vγ4^+^ chain was assessed in WT and Vγ4^−/−^/Vγ6^−/−^ neonatal thymocytes, and further compared with the fetal Vγ4^−/−^/Vγ6^−/−^ thymus at embryonic day (ED) 17.5 (Figure S4B). In the neonatal thymus of Vγ4^−/−^/Vγ6^−/−^ mice, Vγ1 T cells nicely resemble a CD44^high^ IL‐17^+^ phenotype, which is comparable to Vγ4 T cells in WT conditions. In addition, the distribution of CD44^+^ and IL‐17^+^ T cells with respect to the Vγ1‐chain usage in embryonic Vγ4^−/−^/Vγ6^−/−^ thymocytes appears to be consistent with a previous report of Haas et al. [13]. Furthermore, the assessment of IL‐17^+^ cells among Vγ1 T cells in the Vγ4^−/−^/Vγ6^−/−^ embryonic thymus gives evidence for IL‐17 production at this developmental stage (Figure S4C). In summary, subsets of Vγ1 T cells in the adult thymus of Vγ4^−/−^/Vγ6^−/−^ mice share molecular features with developing γδ17 cells. Moreover, Vγ1 T cells produce IL‐17 in the fetal and neonatal thymus of Vγ4^−/−^/Vγ6^−/−^ mice.

## 3 Discussion

The Vγ‐chain of the TCR is commonly used to categorize γδ T cells into different subsets.

Vγ4 and Vγ6 T cells are the dominant IL‐17 producers, although IL‐17 production is not confined to these two subsets [34]. Here, we demonstrate the presence of Vγ1 T cells that secrete IL‐17 in peripheral lymphoid organs of mice lacking Vγ4^+^ and Vγ6^+^ TCRs.

Several studies have demonstrated that Vγ1 T cells produce IL‐17 both at steady state and in disease models. In mice with chronic granulomatous disease, IL‐17‐producing pulmonary γδ T cells expressing a Vγ1^+^ TCR were reported [43]. In *S. aureus*‐infected mice, a minority of renal Vγ1 and Vγ4 T cells demonstrated simultaneous IL‐17A and IFN‐γ production [44]. Our data indicated IL‐17A secretion by Vγ1 T cells in the absence of Vγ4 and Vγ6 T cells; however, the overall IL‐17A production capacity of peripheral γδ T cells was reduced. Consistent with these results, it has been reported that, under steady‐state conditions, some IL‐17^+^ Vγ1 T cells are visible in the thymus, peripheral lymphoid organs, lung, and liver [13]. Another study showed that expression of a monoclonal δ‐chain supports the development of Vγ1 T cells and that some of these cells express IL‐17^45^. Notably, the functionality of γδ T cells being positive for the Vγ6^+^ or Vγ4^+^ TCRs that pair with the monoclonal δ‐chain was not impaired [45]. Overall, there is a connection between the Vγ‐chain and IL‐17‐production capability. Our data support the idea that at least three Vγ‐chains, namely Vγ1, Vγ4, and Vγ6, can induce or support IL‐17 secretion. However, the fact that a large proportion of Vγ4 and Vγ1 T cells do not produce IL‐17 suggests that the Vγ‐chain alone may not be sufficient for IL‐17 induction.

Our scRNA‐seq data from Vγ1 T cells, which were isolated from the lungs of Vγ4^−/−^/Vγ6^−/−^ mice, revealed high expression of molecules involved in TCR signaling, including *Lck*, *Lef1*, and *Tcf7*, within the cluster exhibiting γδ17 cell‐associated gene expression. Notably, *Lck*, *Lef1*, *Tcf7*, and others have been reported to be expressed in IFNγ‐committed cells [23, 35, 41], whereas the TCR activity of γδ17 cells is associated with Syk activity and the phosphoinositide 3‐kinase pathway, which is important for inducing and maintaining the transcriptional program of IL‐17 producers [19].

IL‐17 γδ T cells predominantly develop in the early stages of life, and it is presumed that the early fetal thymus and/or embryonic hematopoietic sources play a key role in their generation [13, 17]. Here, we demonstrate the presence of IL‐17^+^ Vγ1 T cells in the late embryonic and neonatal thymi in Vγ4^−/−^/Vγ6^−/−^ mice. The data presented here are consistent with the presence of Vγ1, Vγ4, and Vγ6 T cells that are IL‐17^+^ and CD44^+^ in WT fetal and neonatal thymus, as reported by Haas et al. [13]. Moreover, IL‐17^+^ Vγ1 T cells and newly generated γδ17 cells were also detected in the adult thymus of Vγ4^−/−^/Vγ6^−/−^ mice. Expression of the key transcription factors for IL‐17 production, c‐Maf and RORγt [40], among developing CD24^+^ Vγ1 T cells in the adult thymus indicates that Vγ1 T cells with the potential to produce IL‐17 are also generated postnatally. A similar concept has been proposed for a subset of IL‐17^+^ Vγ4 T cells [46], and the present results on the TCR repertoires of IL‐17‐secreting cells further support this concept for IL‐17^+^ Vγ1 T cells. When we compared the TCR repertoire, focusing on *Trdv1* and *Trdv5* sequences, of IL‐17^pos^ and IL‐17^neg^ γδ T cells isolated from adult Vγ4^−/−^/Vγ6^−/−^ mice, we found no specific abundance of fetal‐derived clones in either subset. The TCR repertoire of *Trdv1* ^+^ and *Trdv5* ^+^ sequences was highly diverse in Vγ1 T cells, with no specific selection of Vγ1 T cells to become IL‐17 producers. It should be noted that our study was limited by the selection of only two possible paired δ chains based on previous research [33]. Together, these findings demonstrate that IL17^+^ Vγ1 T cells in Vγ4^−/−^/Vγ6^−/−^ mice can be generated both before and after birth.

γδ T cells are known to be the main IL‐17 producers in various tissues such as the skin, lung, or reproductive tract. There, they play a role in infection control and inflammatory diseases [34]. It is speculated that, in early life, fetal‐derived γδ17 cells migrate to the appropriate body location, functionally adapt in the tissues, where they are maintained into adulthood [10]. Future studies may need to address cytokine or chemokine signals that guide the adaptation to and maintenance in peripheral tissues. Additionally, scRNA‐seq analysis of IL‐17^+^ γδ T cells revealed high expression of *Nur77*, indicating TCR signaling [38], particularly in skin γδ T cells, suggesting that the TCR itself may be involved in these processes. Vγ1 T cells were rarely found in the skin of Vγ4^−/−^/Vγ6^−/−^ mice, suggesting that their biology does not support adaptation or maintenance in this tissue niche.

Given the high abundance of Vγ1 T cells in the lungs, we focused primarily on characterizing lung γδ T cells in Vγ4^−/−^/Vγ6^−/−^ mice. The lungs are exposed to various pathogens due to their physiological function of gas exchange. γδ T cells play a role in defending the host against bacteria and viruses. They are also involved in allergic diseases, inflammation, and fibrosis, due to their ability to rapidly produce large quantities of cytokines, primarily IL‐17 and IFN‐γ [47]. Our data suggest that IL‐17^+^ Vγ1 T cells only partially compensate for Vγ4^+^ and Vγ6^+^ IL‐17‐producers in the lymphoid organs and lungs of Vγ4^−/−^/Vγ6^−/−^ mice. Consistent with these results, the decreased IL‐17 production in the KO mice leads to a failed granuloma formation, and hence impaired protection against pulmonary mycobacterial infection, as previously reported [48].

Collectively, in Vγ4^−/−^/Vγ6^−/−^ mice, we observed that Vγ1 T cells exhibit phenotypic and transcriptional profiles resembling those of γδ17 cells, suggesting a compensatory response. While they do contribute to IL‐17 production in the periphery, this is less robust than that of Vγ4 and Vγ6 T cells.

## 4 Material and Methods

### 4.1 Mouse Strains

C57BL/6NCrl and B6.TCR‐Vγ4^−/−^/Vγ6^−/−^ mice were housed under specific pathogen‐free conditions at the central animal facility of Hannover Medical School. B6.TCR‐Vγ4^−/−^/Vγ6^−/−^ mice [27] were kindly provided by Dr. Rebecca L. O'Brien (National Jewish Health, Denver, USA). Mice of both genders were used, randomly allocated to all experiments. Experimental procedures were conducted according to institutional guidelines approved by Lower Saxony State Office for Consumer Protection and Food Safety, Animal Care and Use Committee (reference numbers 17/2704 and 2021/276). Analysis of embryonic thymocytes from B6.TCR‐Vγ4^−/−^/Vγ6^−/−^ mice were conducted at the University of Glasgow, UK, under the licence number PPL number PP0826467.

### 4.2 Isolation of Lymphocytes

Thymus, spleen, and peripheral lymph nodes (pLNs) (including superficial cervical, axillary, brachial, and inguinal LNs) cells were mashed and filtered through nylon gauze to obtain single‐cell suspensions.

To enrich γδ T cells in thymus samples, CD4^+^ and CD8^+^ T cells were depleted using the Streptavidin Nanobeads Protocol. Initially, cells were incubated with anti‐CD4‐biotin (RM4.5) and anti‐CD8a‐biotin (53–6.7) for 30 min, followed by incubation with magnetic Streptavidin Nanobeads (Cat#480016). The magnetically labeled fraction was retained using a magnetic separator, and then the untouched cells were collected for further analysis.

For isolation of lymphocytes from the lung and uterus, the tissues were cut into small pieces and treated with digestion buffer (0.5 mg/mL Collagenase D (Cat#1108886601, Worthington) and 0.025 mg/mL DNase I) at 37°C for 45 min. The digestion was stopped by 20 mM EDTA for 5 min, followed by filtering with a 40 µm pore Cellstrainer. Cells were retrieved via Lympholyte M density gradient centrifugation and re‐suspended in MACS buffer.

The isolation of lymphocytes from ear skin was described before [38]. Briefly, ears were split, cut, and then incubated with digestion buffer (RPMI media supplemented with 2 mg/mL collagenase IV (Worthington) and 187.5 mg/mL DNase I (Roche)) for 75 min at 37°C under 1400 rpm of shaking. Digestion was stopped by adding 5 mM EDTA for 15 min at 37°C under 1400 rpm of shaking, after which the ear tissue was further dissociated using an 18G needle. Cells were then filtered through a 100 mM cell strainer (Falcon) for density gradient centrifugation using 40% and 70% Percoll solutions and finally resuspended in MACS buffer.

### 4.3 Flow Cytometry

Following BD Biosciences antibodies were used: anti‐TCRβ PE‐Cy5 (H57‐597), Cat#553173; anti‐Vγ4 BV480 (UC3‐10A6), Cat#746347; anti‐Vγ1.1 BV711 (2.11), Cat#745456; anti‐CD44 BUV496 (IM7), Cat#741057; anti‐CD24 PE (M1/69), Cat#553262; anti‐IFN‐γ PE‐Cy7 (XMG1.2), Cat#557649; anti‐CD45RB BUV395 (16A), Cat#740211; anti‐CD4 BUV737 (RM4‐5), Cat#612844; anti‐CD8β BUV805 (YTS156.7.7), Cat#755247; anti‐CD27 BV510 (LG.3A10), Cat#563605; anti‐CD3 BV650 (17A2), Cat#569683; anti‐IL‐17A AF700 (TC11‐18H10), Cat#560820. Following eBioscience antibodies were used: anti‐CD8a‐biotin (53‐6.7), Cat#13‐0081‐86; anti‐TCRγ/δ PE (GL3), Cat#15‐5711‐82; anti‐CD44 PE‐Cy7 (IM7), Cat#25‐0441‐82; anti‐CD44 BV421 (IM7), Cat#404‐0441‐82; anti‐CD3e PE‐Cy7 (145‐2C11), Cat#25‐0031‐82; anti‐Vγ4 PE‐Cyanine7 (UC3‐10A6), Cat#25‐5828‐82. ThermoFisher antibodies: anti‐c‐Maf eFluor660, Cat#50‐9855‐82; anti‐RORγt PerCP‐ef710 (B2D), Cat#46‐6981‐82; anti‐IL‐17A APC (TC11‐18H10.1), Cat#53‐7172‐80. Following Miltenyi Biotec antibodies were used: anti‐TCRβ APC‐Vio770 (REA318), Cat#130‐104‐811. Following BioLegend antibodies were used: anti‐CD3 APC‐Fire810 (17A2), Cat#100268; anti‐CD3 FITC (145‐2C11), Cat#100305; anti‐Vγ4 APC (UC3‐10A6), Cat#137708; anti‐Vγ1.1 APC (2.11), Cat#141108; Zombie NIR Fixable Viability Kit, Cat#423106.

For cell surface staining, samples were incubated with 2% FcγR Block antibody (clone 2.4G2) and the respective antibody mixes in FACS Buffer (PBS/3% FCS) for 30 min. All staining procedures were performed at room temperature (RT).

For cytokine staining, cells were initially stained with surface markers, followed by fixation with 150 µL Fixation buffer (Cat#00‐5523‐00, eBioscience) at RT for 30–60 min. Subsequently, the cells were washed and incubated with antibodies prepared in permeabilization buffer at RT for 30 min.

For c‐Maf staining, cells were fixed and permeabilized using the Foxp3 Fixation/PermeabilizationTM Kit according to the supplier's manual (eBioscience). Dead cells were detected by staining with Zombie NIR (Biolegend).

Cells were acquired using a Cytek Aurora spectral flow cytometer (Cytek), and data were analyzed using FlowJo 10.0 software.

### 4.4 In Vitro Cell Stimulation

γδ T cells were enriched from single‐cell suspensions using Mojo Sort magnetic beads (Cat#480016, BioLegend) to deplete CD4^+^ and CD8^+^ T cells, as described above. The cells were then incubated overnight with complete medium (RPMI 1640 medium supplemented with 10% fetal bovine serum (FBS), 1% penicillin‐streptomycin, 1% nonessential amino acids (NEAA), 0.001% β‐mercaptoethanol, 1% HEPES, and 1% sodium pyruvate) in the presence of murine IL‐1β (50 ng/mL, Cat#211‐11B, PeproTech) and IL‐23 (50 ng/mL, Cat#1887‐ML‐010, R&D)). Subsequently, the cells were re‐stimulated with PMA (50 ng/mL, Cat#524400, Sigma‐Aldrich) and Ionomycin (2 µg/mL, Cat#I‐24222, Invitrogen) and incubated for 3 h at 37°C with Brefeldin A (10 µg/mL, Cat#B6542‐5MG, Sigma‐Aldrich), followed by flow cytometric analysis.

### 4.5 Isolation of IL‐17 Producing Γδ T Cells for TCR‐seq

The mouse IL‐17 Secretion Assay Detection Kit (Cat#130‐094‐207) from Miltenyi Biotec was used according to the manufacturer's instructions. For stimulation, pLN cells from Vγ4^−/−^/Vγ6^−/−^ knockout mice were resuspended in culture medium with Ionomycin (1 µg/mL) and PMA (10 ng/mL) and incubated for 3 h at 37°C. For isolation by FACS sorting, the anti‐Biotin‐APC, anti‐TCRγ/δ AF488 (GL3), anti‐TCRβ PE‐Cy5 (H57‐597), and anti‐CD3e PE‐Cy7 (145‐2C11) antibodies, as well as Zombie NIR (Biolegend), were used. Cell sorting was carried out on the FACS‐Aria Fusion machine (Becton Dickinson BD Biosciences).

### 4.6 TCR‐seq

mRNA extraction was performed from FACS‐sorted IL‐17^+^ and IL‐17^−^ pLN γδ T cells according to the instructions of the RNeasy mini kit (Qiagen). For *Trd* (Trdv1 and Trdv5) repertoire analysis, the isolated mRNA was reverse‐transcribed using SMARTer RACE 5′‐3′ PCR Kit (Clontech) with a customized protocol. Next, the CDR3 region of Trd clones was amplified via RACE PCR. For all conditions and to control PCR contamination, negative controls (H2O) were run together with each PCR and also subjected to sequencing. All PCR amplicons were subsequently prepared for paired‐end Illumina sequencing (v2 500 cycles), using the Illumina MiSeq platform according to the Illumina guidelines. Sequencing reads from obtained FASTQ files were aligned and annotated with MiXCR according to the international immunogenetics information system [49]. Annotated files were analyzed with V(D)J tools [50] and the Immunarch package in R.

### 4.7 scRNAseq Library and Bioinformatics Flow

Lung Vγ1^+^ T cells from Vγ4^−/−^/Vγ6^−/−^ mice (KO) and lung non‐Vγ1 T cells (mainly Vγ4 and Vγ6 T cells) from WT mice (WT) were FACS sorted. Cells were subjected to scRNAseq library generation using the Chromium Next GEM Single Cell V(D)J Reagent Kits v1.1 according to the manufacturer's protocol (10xGenomics). The scRNAseq libraries were sequenced with the Illumina NexSeq 500 platform. Sequence reads were aligned to the reference mouse genome mm10 (UCSC), and cell barcode‐gene matrices were counted with Cell Ranger 3.1 (10x Genomics). The cell barcode‐gene matrices were then processed with Seurat v4.3.3 under R v4.0.3 to remove low‐quality cells (genes >200, Features <4000, % mitochondrial genes <10). The two datasets were merged and followed by normalization and scaling, dimension reduction, and clustering. Aggregated lineage marker scores were computed via “AddModuleScore” and depicted with “FeaturePlot”. The “FindMarkers” function compared differential expression gene (DEG) between different groups, with a focus on genes observed in at least 10% of cells and maintaining a logFC threshold of 0.5.

### 4.8 Statistics

All statistical analyses were performed using GraphPad Prism version 9.5.0. For comparisons between two groups, nonparametric unpaired Mann–Whitney *U* tests were used. For comparisons among three or more groups, the nonparametric Kruskal–Wallis test followed by Dunn's multiple comparisons correction was applied. Data are presented as mean ± SEM, and *p*‐values < 0.05 were considered statistically significant. The following thresholds were used to indicate significance (ns *p* > 0.05, *p < 0.05, **p < 0.01, ***p < 0.001, ****p < 0.0001).

## Author Contributions

Ziqing Wang performed experiments, analyzed data, and wrote the manuscript. Tao Yang analyzed data and contributed to writing the manuscript. Lara‐Marie Behrens, Federico Lupo, and Seth B. Coffelt performed experiments. Anika Janssen performed experiments. Immo Prinz and Inga Sandrock designed the study. Sarina Ravens designed the study, supervised the study, and wrote the manuscript.

## Ethics Statement

Experimental procedures were conducted according to institutional guidelines approved by Lower Saxony State Office for Consumer Protection and Food Safety, Animal Care and Use Committee (reference numbers 17/2704 and 2021/276). Experiments to study fetal thymocytes were conducted at the University of Glasgow, UK, under the license number PPL number PP0826467.

## Conflicts of Interest

The authors declare no conflicts of interest.

## Peer Review

The peer review history for this article is available at https://publons.com/publon/10.1002/eji.70061.

## Supporting information




**Supporting Information file 1**: eji70061‐sup‐0001‐SuppMat.pdf

## Data Availability

Raw single‐cell sequencing and TCR sequencing data are available under NCBIs Gene Expression Omnibus (GSE284167 and GSE284198).
