# Supplementary material for: Partial Compensation of IL‐17 Production by Vγ1 T Cells in the Absence of Vγ4 and Vγ6 T Cells
Source: Eur J Immunol. 2025 Sep 20;55(9):e70061. doi: 10.1002/eji.70061 (PMC12449965; doi:10.1002/eji.70061)
Supplement: Supplementary file 1 — Supporting Information file 1: eji70061‐sup‐0001‐SuppMat.pdf [file EJI-55-e70061-s001.pdf]

Figure S1.

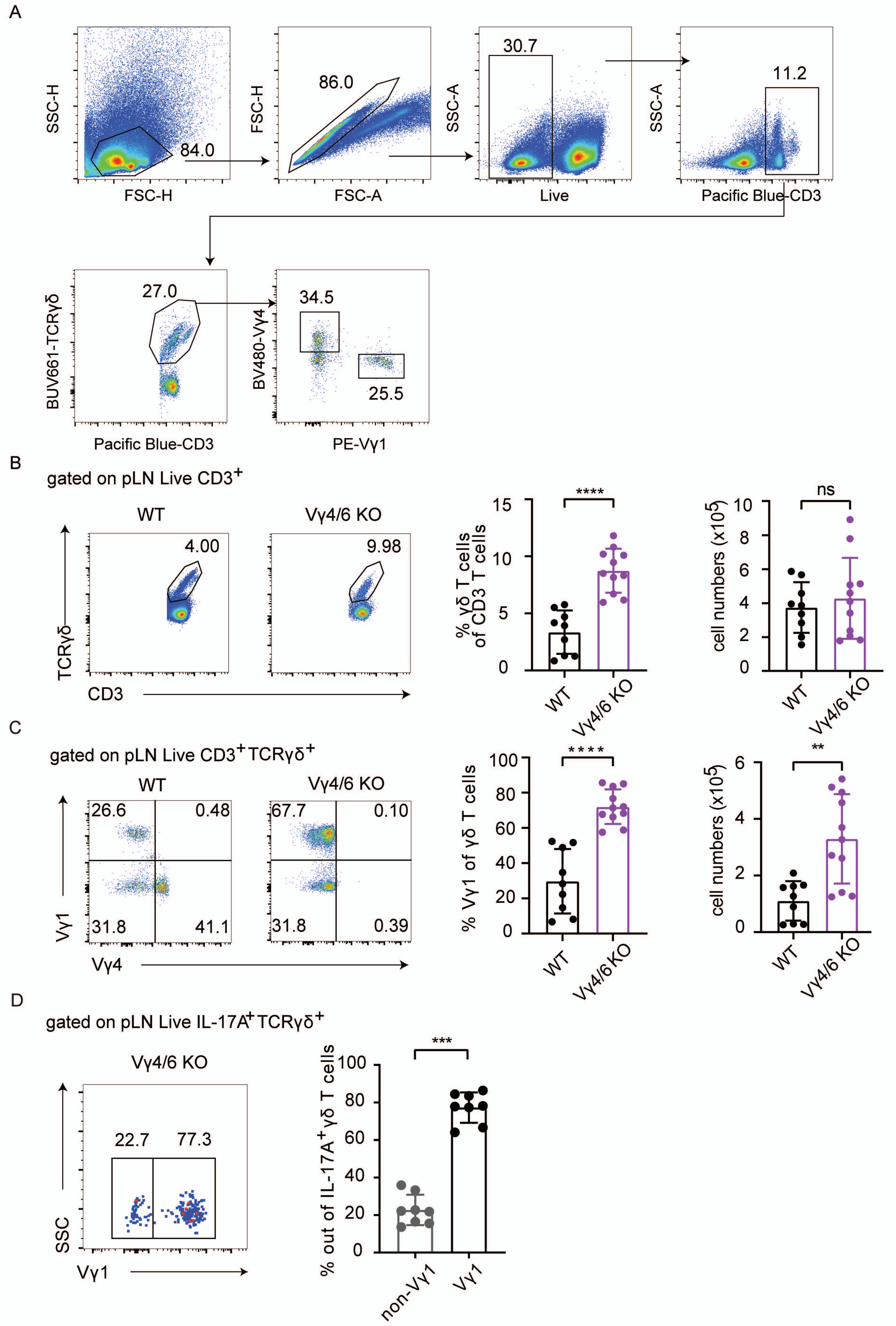

Figure S1: Analysis of pLN γδ T cells in in WT and Vγ4−/−/Vγ6−/− adult mice. (A) Representative gating strategy for the analysis of pLN γδ T cells by flow cytometry. (B) Representative FACS plots and graphs show the frequency and cell numbers of γδ T cell in pLNs of WT and Vγ4$^{-/-}$/Vγ6$^{-/-}$ mice. (C) Representative FACS plots and graphs show the frequency and cell numbers of Vγ1 T cells in pLNs of WT and Vγ4$^{-/-}$/Vγ6$^{-/-}$ mice. (D) Vγ1 and non-Vγ1 T cell frequencies of IL-17A^{+} γδ T cells in the pLNs of Vγ4$^{-/-}$/Vγ6$^{-/-}$ mice. Data are representative of two or three independent experiments, with n = 3–4 mice per group. P values were determined using an unpaired Mann-Whitney test (ns $p > 0.05$, * $p < 0.05$, ** $p < 0.01$, *** $p < 0.001$, **** $p < 0.0001$). γδ T = gamma-delta T cells; PLNs = peripheral lymph nodes; WT = wild-type; KO= Vγ4$^{-/-}$/Vγ6$^{-/-}$.

Figure S2.

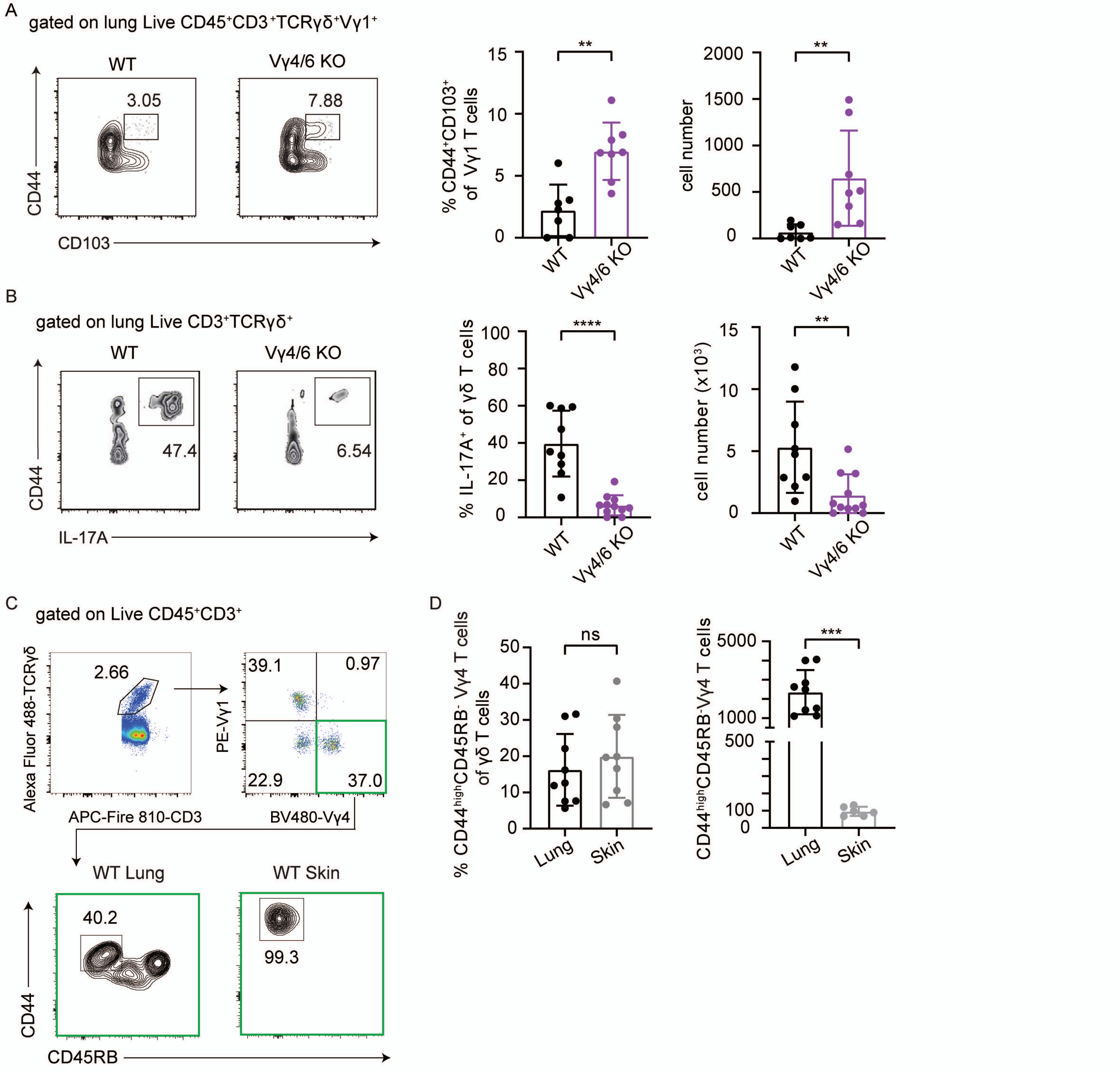

Figure S2: Analysis of tissue γδ T cells in WT and Vγ4$^{-/-}$/Vγ6$^{-/-}$ adult mice. (A) Representative FACS plots and graphs show frequencies and cell numbers of CD44^{+} CD103^{+} Vγ1 T cells in the lungs of WT and Vγ4$^{-/-}$/Vγ6$^{-/-}$ mice. (B) Frequency and absolute number of IL-17A^{+} γδ T cells in the lungs of WT and KO mice after overnight in vitro stimulation with IL-23 and IL-1β. (C) Representative flow cytometry plots show the expression of CD44 and CD45RB on Vγ4 T cells from WT lung and skin. (D) Frequency of CD44^{high}CD45RB^{-} Vγ4 T cells among total γδ T cells, and absolute numbers of CD44^{high}CD45RB^{-} Vγ4 T cells. Data are representative of two or three independent experiments, with n = 3–4 mice per group. P values were determined using an unpaired Mann-Whitney test (ns p > 0.05, * p < 0.05, ** p < 0.01, *** p < 0.001, **** p < 0.0001). γδ T = gamma-delta T; WT = wild-type; KO= Vγ4$^{-/-}$/Vγ6$^{-/-}$.

Figure S3.

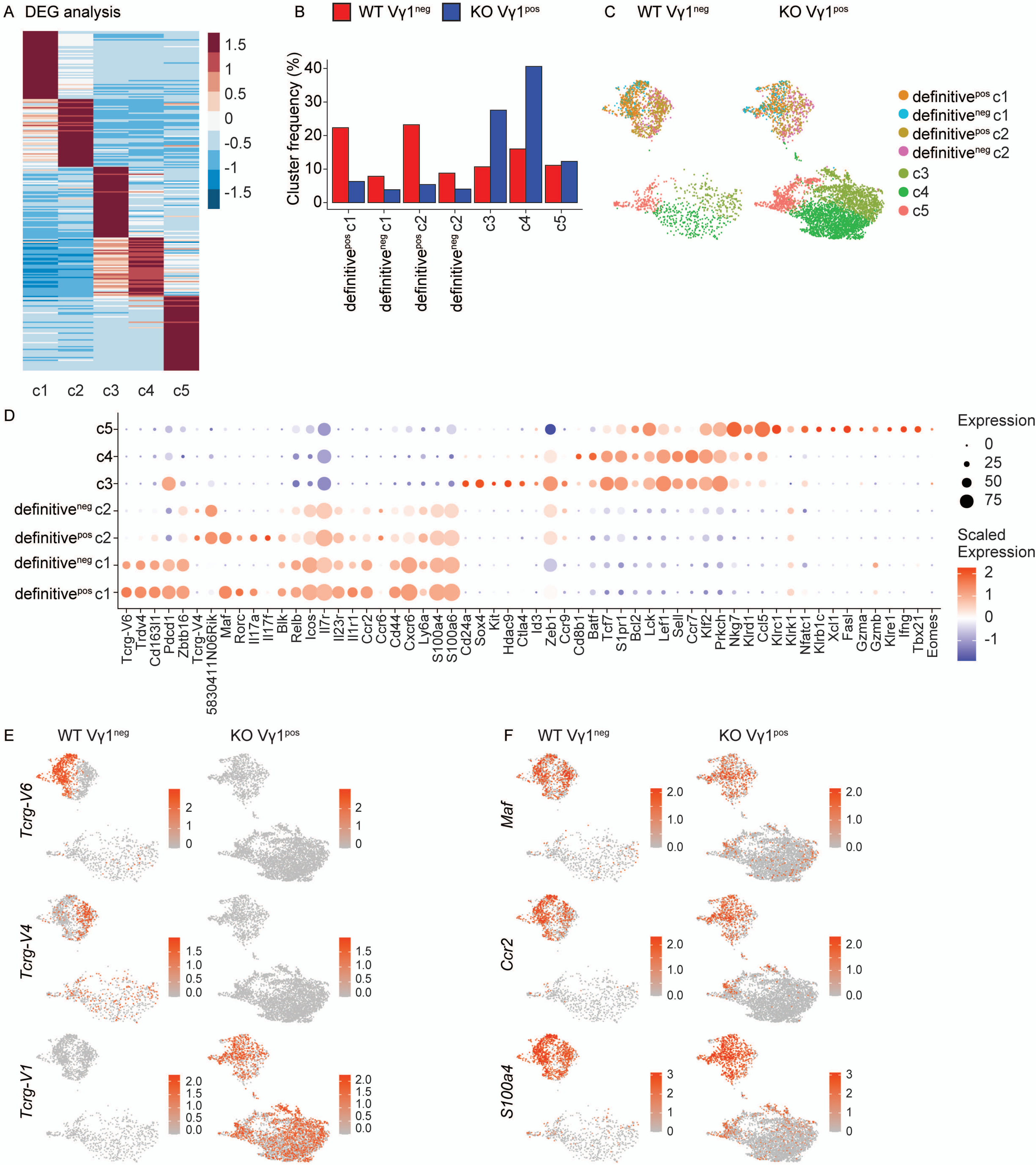

Figure S3: scRNA-seq analysis of Vγ1⁻ T cells from WT lung and Vγ1⁺ T cells from Vγ4⁻/⁻/Vγ6⁻/⁻ (KO) lung. (A) Heatmap represents the top 50 DEGs for each cell Cluster. (B) The bar plot reveals fractions (%) of absolute cell numbers from WT Vγ1neg and KO Vγ1pos T cells that contribute to all clusters. (C) UMAP representation of WT Vγ1neg and KO Vγ1pos T cell transcriptomes, colored by cluster. (D) Dot plots display average gene expression per cluster. Gene expression values were scaled to a log2 fold change (logFC). Dots are colored by average logFC and sized by the percentage of cells expressing the respective gene per cluster.
(E-F) UMAPs show the selected gene expression of WT Vγ1⁻ and KO Vγ1⁺ T cells.

Figure S4.

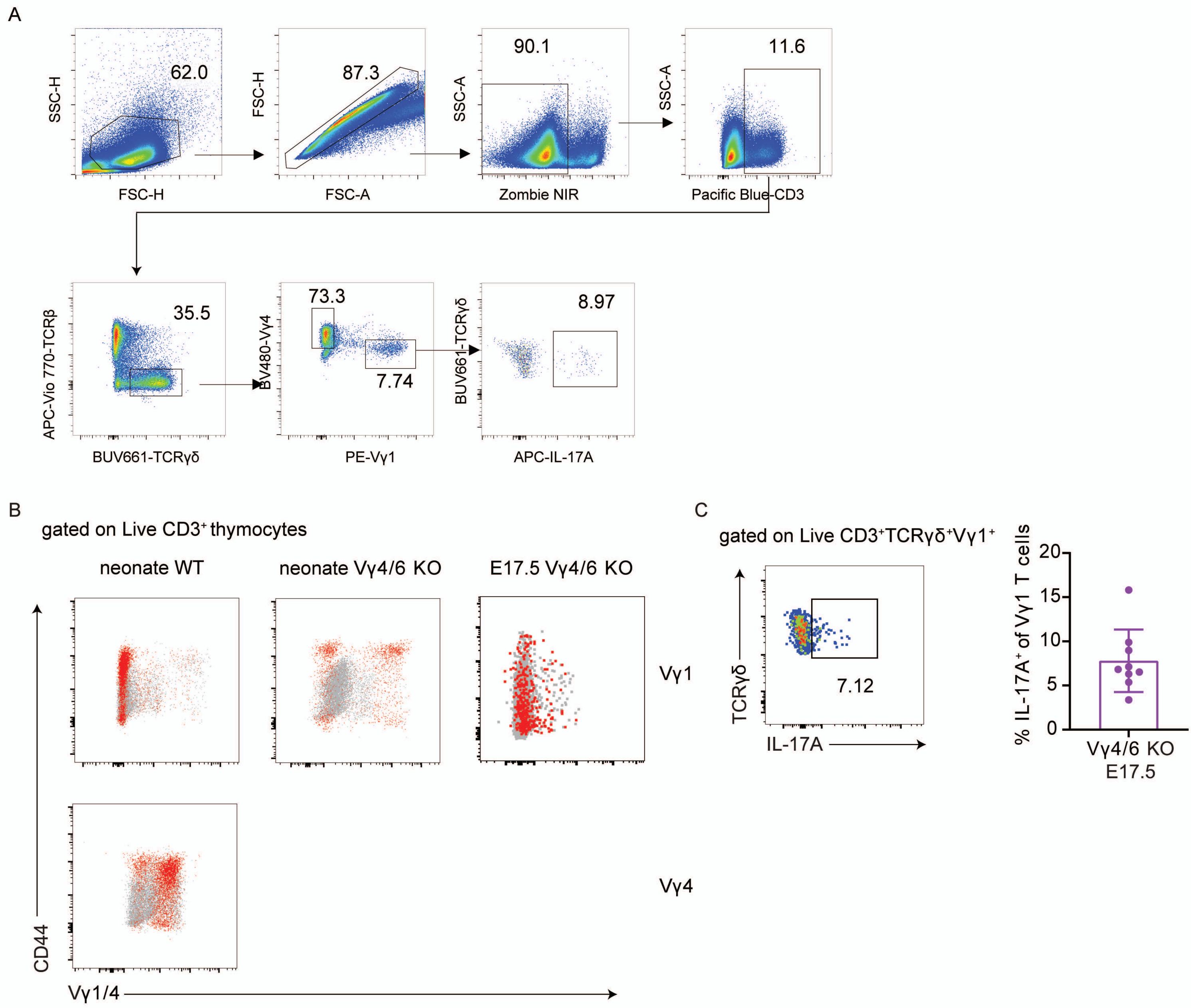

Figure S4: Analysis of thymic γδ T cells in WT and $V\gamma4^{-/-}/V\gamma6^{-/-}$ mice. (A) Representative gating strategy for the analysis of cytokine production by WT neonatal thymic γδ T cells using flow cytometry. (B) Overlay plots show gated $IL\text{-}17^+$ cells (red) in comparison to all $CD3^+$ cells (gray) at different age (E17.5 and postnatal day5) from WT and $V\gamma4^{-/-}/V\gamma6^{-/-}$ mice in a staining with CD44 versus the respective Vγ chains (Vγ1, Vγ4). (C) Representative FACS plots (left) and statistic graph (right) show the frequency of $IL\text{-}17^+$ Vγ1 T cells in the embryonic thymus of $V\gamma4^{-/-}/V\gamma6^{-/-}$ mice. This experiment was performed once; each dot represents one mouse.
